# ECDC call for expression of interest for editing and proofreading, and rapporteur services

**DOI:** 10.2807/1560-7917.ES.2019.24.38.1909191

**Published:** 2019-09-19

**Authors:** Eurosurveillance editorial team

**Affiliations:** 1 European Centre for Disease Prevention and Control (ECDC), Stockholm, Sweden

The European Centre for Disease Prevention and Control (ECDC) invites candidates to express their interest in one or more of the following services:

 

- SUB LIST 1 Rapporteurs services;
- SUB LIST 2 Editing and proofreading services for the scientific journal *Eurosurveillance*;
- SUB LIST 3 Editing and proofreading services for ECDC technical and scientific reports.

 

The deadline expires on 7 May 2023.

For more information, visit the European Union (EU) institutions' eProcurement platform: https://etendering.ted.europa.eu//cft/cft-display.html?cftId=5314

